# The One-stop trial: Does electronic referral and booking by the general practitioner (GPs) to outpatient day case surgery reduce waiting time and costs? A randomized controlled trial protocol

**DOI:** 10.1186/1471-2482-8-14

**Published:** 2008-08-11

**Authors:** Knut Magne Augestad, Arthur Revhaug, Barthold Vonen, Roar Johnsen, Rolv-Ole Lindsetmo

**Affiliations:** 1 Norwegian Centre for Telemedicine, Norway; 2 Department of Gastrointestinal Surgery, University Hospital of North Norway, Norway; 3 Institute of Clinical Medicine, Tromsø University, Norway; 4 Institute of Public Health and General Practice, Norwegian University of Science and Technology, Trondheim, Norway; 5 University Hospitals, Case Medical Center, Cleveland, Ohio, USA

## Abstract

**Background:**

Waiting time and costs from referral to day case outpatient surgery are at an unacceptably high level. The waiting time in Norway averages 240 days for common surgical conditions. Furthermore, in North Norway the population is scattered throughout a large geographic area, making the cost of travel to a specialist examination before surgery considerable. Electronic standardised referrals and booking of day case outpatient surgery by GPs are possible through the National Health Network, which links all health care providers in an electronic network. New ways of using this network might reduce the waiting time and cost of outpatient day case surgery.

**Materials and Methods:**

In a randomised controlled trial, selected patients (inguinal hernia, gallstone disease and pilonidal sinus) referred to the university hospital are either randomised to direct electronic referral and booking for outpatient surgery (one stop), or to the traditional patient pathway where all patients are seen at the outpatient clinic several weeks ahead of surgery.

Consultants in gastrointestinal surgery designed standardised referral forms and guidelines. New software has been designed making it possible to implement referral forms, guidelines and patient information in the GP's electronic health record. For "one-stop" referral, GPs must provide mandatory information about the specific condition. Referrals were linked to a booking system, enabling the GPs to book the hospital, day and time for outpatient surgery. The primary endpoints are waiting time and costs. The sample size calculation was based on waiting time. A reduction in waiting time of 60 days (effect size), 25%, is significant, resulting in a sample size of 120 patients in total.

**Discussion:**

Poor communication between primary and secondary care often results in inefficiencies and unsatisfactory outcomes. We hypothesised that standardised referrals would improve the quality of information, making it feasible to use a one-stop approach for all patients undergoing surgery on an outpatient basis for inguinal hernia, pilonidal sinus and gallstones.

In this study we wanted to investigate the waiting time and cost-effectiveness of direct electronic referral and booking of outpatient surgery compared to the traditional patient pathway, where the patient is seen at the outpatient clinic prior to surgery.

**Trial registration:**

This trial has been registered at ClinicalTrials.gov. The trial registration number is: NCT00692497

## Background

The shift from inpatient to outpatient care is drawing attention to the role of the outpatient clinic and day case surgery. An increased focus on cost benefit and waiting time for examination and treatment of surgical patients has made it necessary to develop new strategies for referral routines and the patient flow between primary and secondary care. One-stop day case surgery, i.e. day case surgery based upon standardised referrals, may be a solution for selected surgical patients [1,2].

In Norway, all patients with an inguinal hernia, gallstones, or pilonidal sinus are primarily examined by a general practitioner (GP). The GP performs a regular clinical examination, and they refer the patient to examination at the surgical outpatient clinic, at the same time forwarding supplemental clinical information about the patient (i.e. age, sex, social status, other diseases and clinical conditions, medication). The referrals are not standardised. Within three weeks, a hospital doctor must read the referral and draw up a treatment or investigation plan. A time schedule for the outpatient clinic examination is proposed on the basis of the information in the referral and the availability of appointments for examinations. All patients are then examined by a surgeon at the outpatient clinic, who in turn refers the patient to outpatient surgery. This in-hospital referral must be read and prioritised by a senior hospital surgeon within two weeks before a final schedule for an operation for the patient is decided.

According to the Norwegian Directorate for Health and Social Affairs, patients with inguinal hernia must wait up to 22 weeks for an examination by a gastrointestinal surgeon at a surgical outpatient clinic, and up to 28 weeks for day case outpatient surgery (Table 1) [3]. Similar or even longer waiting times apply to conditions such as symptomatic gallbladder stones and pilonidal sinus.

**Table 1:** Waiting time reported by the Norwegian Directorate for Health and Social Affairs for surgical outpatient examination and surgery in Norway, February 2008 [3].

	Outpatient Consultation (weeks)	Outpatient Surgery (weeks)
Gallstones	2–22	2–30
Inguinal hernia	2–22	2–28
Pilonidal sinus	2–30	2–48

The newly established Norwegian National Health Network [4] has given us the potential to improve the communication between levels of the health care providers. The National Health Network is intended to support exchange of information and enable affiliated organisations to offer professional support, medical services and administrative services in the network. From a single connection point, users should be able to communicate with all the other participants connected to the network.

The main objectives of the present study were to assess whether standardised electronic referrals based on guidelines and booking of outpatient surgery by the GPs would decrease waiting time and increase the cost-effectiveness of day case outpatient surgery for patients with inguinal hernia, pilonidal sinus and gallstone disease.

## Methods and Design

### Study design

**Figure 1:**
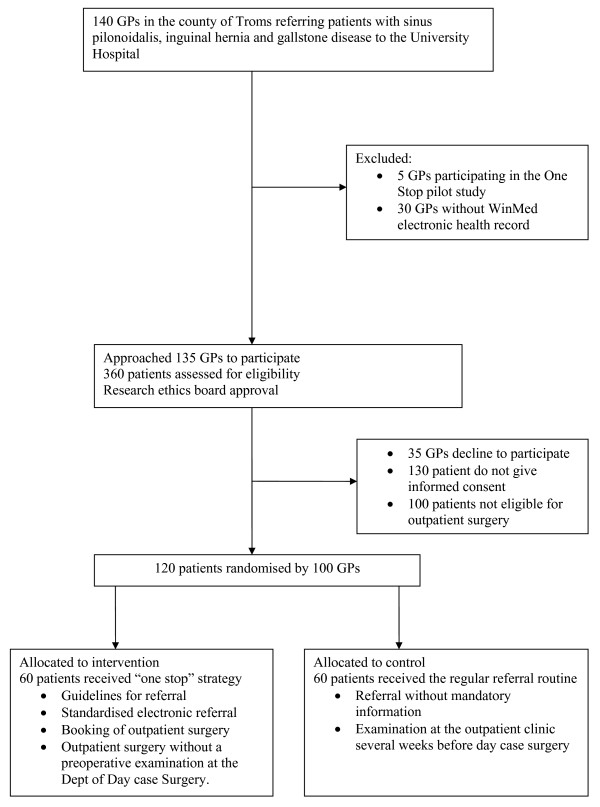
Trial flow chart.

This study is a randomised controlled trial, where patients are randomised at the time of referral either to one-stop strategy (intervention group) or to the regular patient pathway where patients are examined at the surgical outpatient clinic before day case outpatient surgery (control group) (Figure 1).

#### Participants

##### Software producer

**Figure 2:**
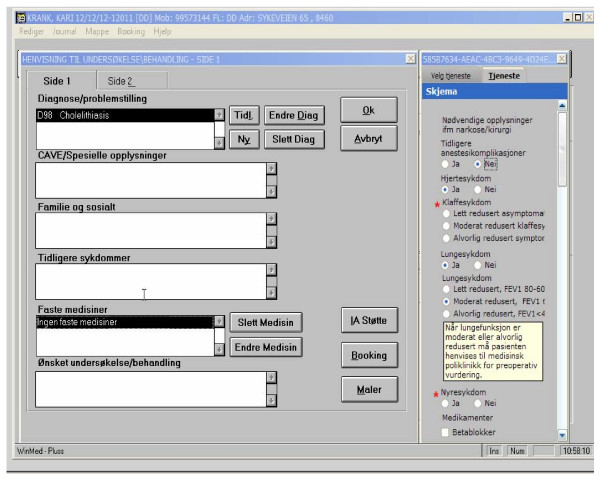
**Beta version of the standardised referral software in the "one-stop" trial**. The software is integrated in the GP's electronic patient health records. The referral forms consist of 3 parts: Information needed for anaesthesia (left side of screen), information needed for surgeons (left side of screen) and additional information provided by the GP (right side of screen). When the GP enters an ICPC code (in this example D98 Cholelithiasis) the left side of the screen appears automatically. Booking of surgery is also done from this screen. This referral is fully integrated in the electronic health record (Profdoc Winmed).

**Figure 3:**
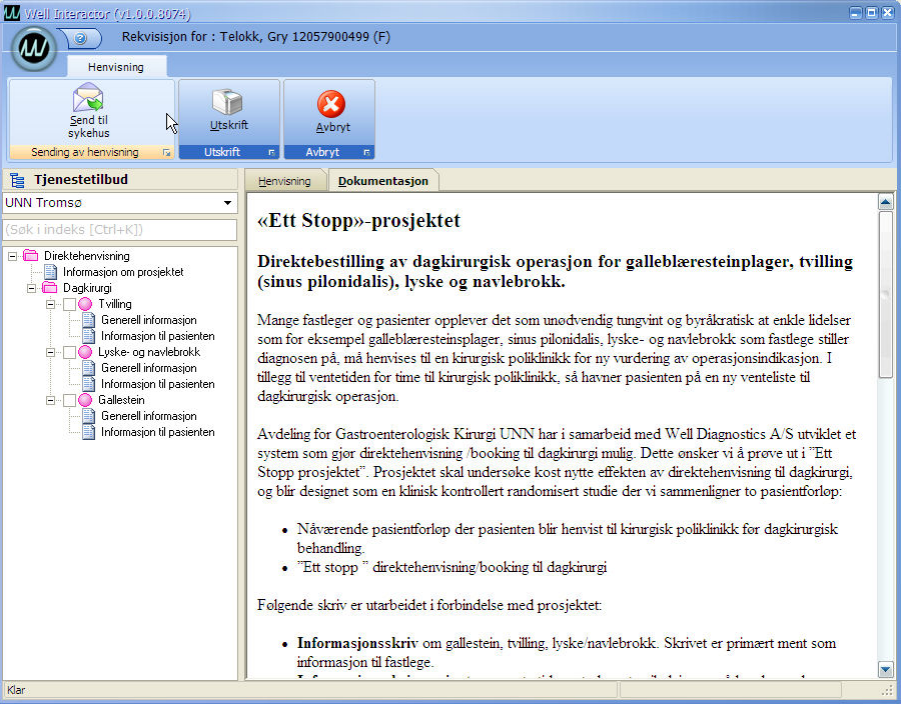
**Beta version of the guidelines for referral in the "one-stop" trial**. Guidelines for referral and information to the patient are integrated in the GP's electronic health record linked to the referral screen. The GP can switch between the referral form and guidelines in the referral process. The guidelines consist of 2 parts: information to the GPs and information to the patient.

The software producer has been involved in several projects involving electronic communication between primary care and secondary care in cooperation with the University Hospital. The software used in the study was developed during a one year-period (Figure 2 and 3). The software for referral and booking will be integrated in the electronic health record in primary and secondary care. For practical and economic reasons, this study includes only GPs who use the electronic health record Profdoc Winmed.

##### General Practitioners

In the county of Troms there are 31 community GP practices with 140 GPs. All these GP practices are connected to the Norwegian National Health Network. Approximately 80% of these GPs are using the electronic health record ProfdocWinmed. Guidelines and referral forms will be implemented in this electronic health record during a three-month period for all GPs who agree to participate in the trial.

##### Department of Digestive Surgery at a University Hospital

The University Hospital is located at three different geographical sites, with one large teaching university clinic and two local hospitals. The Department of Digestive Surgery is divided into clinics located at the different hospitals. All these clinics perform the outpatient surgical procedures included in the trial (Pilonidal sinus: Bascom plasty. Inguinal hernia: Lichtenstein repair. Gallstone disease: laparoscopic cholecystectomy)

##### Patients

Patients are eligible if they have been diagnosed by their GP for an inguinal hernia, sinus pilonoidalis or gallstone disease requiring surgical treatment. Patients with medical conditions making them unfit for outpatient surgery who are admitted to the surgical department prior to surgery are not eligible. In 2007, 366 patients were surgically treated at the Day Case Surgical Department for these three conditions. Patients will be included after giving written informed consent.

##### Intervention

The one-stop strategy is a set of interventions directed at GPs referring patients to the University Hospital. The interventions include: Guidelines for referral, standardised electronic referrals, booking for outpatient surgery and a patient information form.

Guidelines for referral: Guidelines for inguinal hernia, sinus pilonoidalis and gallstone disease were developed (Figure 3). All surgeons performing the surgical procedures reviewed the giudelines, and after a three-month period consensus was reached. The guidelines consisted of signs and symptoms for the condition, indications and contraindications for surgery, necessary blood samples and radiological examinations prior to surgery (for gallstone disease). Photographs of the condition were included in the guidelines for sinus pilonoidalis and inguinal hernia. These guidelines were implemented in the GPs electronic health record.

Standardised referral forms: Standardised referral forms were developed by specialists in gastrointestinal surgery in cooperation with GPs (Figure 2). These referral forms consisted of three parts: information needed for anaesthesia (mandatory), information needed for surgeons (mandatory) and additional information provided by the GPs. These referral forms were implemented in the GPs' electronic health records (Profdoc Winmed).

Booking of time, date and hospital for surgery: The booking system is integrated in the electronic health record (Profdoc Winmed); booking is done at the same time as referral. Patients can choose the optimal time and hospital for surgery. The system is managed by the Day Case Surgical Department. Available times and locations for surgery during a 6-month period are presented. Surgical capacity that has not been booked within two weeks of the relevant date is withdrawn and offered to patients who are not participating in the trial.

Patient information form: A patient information form (printable, integrated in the electronic health record) was prepared by a specialist in gastrointestinal surgery. The form included information about the place and time for surgery, information about the surgical procedure, and advice for the patient following surgery. Information about possible complications of surgery was also provided.

#### Control arm

The control group consists of patients randomised to use the regular patient pathway prior to day case outpatient surgery. All these patients are referred to the surgical outpatient clinic. At the outpatient clinic patients are examined by a surgeon, who determines whether surgery is indicated. If so, patients are referred to outpatient surgery and the surgical procedure is performed several weeks after the examination.

#### Objectives

We wish to test the efficacy of the one-stop strategy in decreasing waiting time and increasing the cost-effectiveness of selected day case surgical outpatient procedures. We hypothesise that patients exposed to the one-stop strategy (intervention) will have decreased waiting time and increase the cost-effectiveness for the hospital, compared to patients who use the regular patient pathway, i.e. examination at the surgical outpatient clinic prior to outpatient surgery.

#### Outcome measures

##### Primary outcome

##### Waiting time for outpatient surgery

This time interval will be calculated retrospectively and compared between the two groups. The waiting time specified by health authorities is unreliable (Table 1); waiting time must therefore be calculated separately for each individual using dates from the electronic health record. This will consist of the following dates: the date the referral is noted in the hospital electronic health record, the date of outpatient examination (for the control group) and the date of outpatient surgery.

##### Cost-minimisation analysis

We will use a cost-minimisation analysis in this study, since this health care intervention has a similar medical outcome for both groups, i.e. surgical outpatient treatment [5]. The analysis will have a public health provider perspective. This includes travel costs, since the public health service pays for these costs in Norway. Direct health care costs that are the same in both alternatives will be excluded. The costs for the GPs providing this service will include investment, support, and time costs. Costs of traditional referral will include hospital administration, examination at the Surgical Outpatient Department (surgeon time, nurse time), surgical treatment at the Department of Day Case Surgery (surgeon time, anaesthetist time, and nurse time) unused surgical capacity due to incorrect referral, and patient travel costs. All costs will be estimated separately and compared between the two groups.

We will then conduct a threshold analysis. This analysis calculates the number of patients needed to break even, i.e. where the cost of providing a one-stop service equals the cost of traditional referrals.

This value is given by:

Total cost of one-stop service = Total cost of traditional referral

F_OS _+ xV_OS _= F_TR _+ xV_TR_

X = (F_OS _+ F_TR_)/(V_OS _- V_TR_)

X = number of consultations; F = fixed costs; V = variable costs; OS = one-stop; TR = traditional referral;

##### Secondary outcome

All GPs participating will be posted a questionnaire when all patients have been included. This questionnaire will primarily be designed to evaluate the GPs. satisfaction with the new software, focusing on areas such as user friendliness and time consumption.

##### Logistics

For patients who are included, the GP must follow certain steps to avoid trial bias. However, it is important that the procedures for enrolling patients in the trial are not so time-consuming that GPs choose not to include patients. The steps in the inclusion process will be:

1. Identify patients who meet the inclusion criteria according to guidelines provided in the electronic health record.

2. Obtain written informed consent from all eligible patients.

3. Call the randomisation centre at the University Hospital. The patient is then registered in the study database and thereby automatically randomised to either the one-stop approach or the traditional patient pathway.

4. If the patient is randomised to the intervention group, the standardised referral forms provided by the electronic health record must be used. If the patient is randomised to the control group, the traditional referral forms (also provided y the electronic health record) with no mandatory information regarding the specific disease must be used.

5. If randomised to the intervention group: book place, time and date of surgery

6. Print the information form about the surgical procedure and where and when to meet for surgery and give to the patient.

##### Sample size

To calculate the sample size needed, we identified all patients who had undergone the specified surgical procedures within three months of normal surgical activity (September, October and November 2007). Of these 33 patients, 22 patients could be included in a one-stop protocol. The rest of the patients were not eligible due to different patient pathways.

We calculated the average waiting time from referral to examination at the surgical outpatient dept, the waiting time from outpatient examination to surgery, and the total waiting time (Table 2).

**Table 2:** Mean waiting time for examination and surgical treatment for selected day case prcedures at a University Hospital during a three-month period of normal activity.

	GP referral to examination at the surgical outpatient clinic (days)	Internal hospital referral to day case surgery (days)	Total waiting time (days)
Waiting time^a ^(mean)	122	119	241
Standard deviation	118	92	154

^a ^Inguinal hernia, pilonidal sinus and uncomplicated gallstone disease

Based upon calculations in Table 2, we assume that there is no significant difference in waiting time between the three groups. Alpha and beta were set at 0.05 and 0.2 respectively. We estimate that a significant decrease in mean waiting time for surgery would be 60 days. The standard deviation is estimated as 120 days. We assume an exclusion rate of 20%, so that a sample size of 60 patients in each group is required.

#### Randomisation

Patients will be randomised at the time of referral by the GP. The randomization service is telephone-based and managed by the University Hospital. Patients are stratified into three groups according to their diagnosis.

#### Blinding

To avoid bias, surgeons and administrative personnel are blinded to patients' status in the trial. Referrals of pilonidal sinus, gallstone disease and inguinal hernia to the surgical outpatient clinic will be examined by the principal investigator. The examining and operating surgeon at the surgical department will be blinded to all data concerning the one-stop trial. Patients enrolled in the trial will be mixed with patients referred to the surgical outpatient department by GPs not participating in the trial.

#### Data gathering

Data are collected in the intervention group and control group in identical ways. Outpatient surgery is performed on a weekly basis, therefore hospital charts will be reviewed weekly. The study has been approved by the Norwegian Data Inspectorate. All data will be handled with strict confidentiality, and study reports or presentations will maintain the anonymity of patients, surgeons, GPs and hospitals. Data collection will be complete by the end of 2009.

#### Analysis

Treatment arms will be compared with respect to potential covariates using continuous and categorical variables. This will include variables related to patients (sex, age, type of disease, date of referral, date of outpatient examination, date of outpatient surgery), cost (patient sick leave, GP examination, hospital administration, surgical outpatient examination, surgical outpatient treatment, unused surgical capacity due to incorrect referral, patient travel cost). The results will be presented as intention-to-treat analyses and treatment analyses.

We will conduct analyses using the latest version of SPSS. The trial will be reported according to the CONSORT standards for reporting randomised trials [6,7].

The results will be expressed as an odds ratio (for binary outcomes), hazard ratio (time to event outcomes) or mean difference (for continuous outcomes) with corresponding standard errors, 95% confidence intervals and associated p-values. For all two-sided tests we use alpha = 0.05 level of significance.

#### Ethics

The Regional Committee for Medical Research Ethics, North Norway approved this protocol (P REK NORD 122/2006). Patients must provide written informed consent before entering the trial.

## Discussion

The one-stop trial will assess the one-stop strategy, which is designed to decrease waiting time and improve cost-effectiveness for surgical outpatient treatment.

Two studies have assessed a one-stop approach for surgical conditions involving the abdominal wall, pilonidal sinus, soft-tissue tumours, or proctologic disease [1,2].

In a Spanish study the delay from referral until surgery was reduced by 60% and the number of trips for appointments was reduced by 66.6% [1]. Because of its feasibility, acceptability, and cost-efficiency, the direct referral system has the potential to improve relations between primary and specialised care and enhance the quality of care by shortening the delay to treatment. In a study from 2004, patients were sent an appointment for "one-stop" inguinal hernia treatment [2]. It was concluded that patients with unilateral primary inguinal hernias can be seen, assessed and treated on the same day. One-stop inguinal hernia surgery reduces the number of patient visits to the hospital and could be expanded to incorporate many more hernia repairs and other day case procedures.

Other surgical conditions have also been shown to be suitable for improved referral routines, communication and patient logistics between the primary and secondary interface. Better communication between referral centres and GPs combined with continuing medical education programmes may be useful tools to improve appropriate management for surgical patients with faecal incontinence [8]. One-stop clinics have been reported to improve examination and treatment of patients with head and neck lumps [9,10]. Rectal bleeding clinics can facilitate early diagnosis of colorectal malignancy and can also provide a "one-stop shop" for treating benign anorectal conditions [11-14]. A recent study [15] is presenting the "Lower Gastrointestinal Electronic Referral Protocol" which was developed to be used alongside the national Choose and Book programme [16]. A dedicated referral protocol addressing all colorectal symptoms would significantly reduce delays in patient pathways with 'straight to test' in secondary care [15].

A general-practice-led model of integrated care can significantly reduce outpatient attendance while improving patient experience for patients with menorrhagia [17,18]. For patients with lower urinary tract symptoms and hematuria, a guideline-based open-access investigation service streamlined the process of outpatient referral and resulting in a decrease in waiting time and fewer outpatient investigations [19]. For patients with breast lumps, a one-stop clinic approach and referral guidelines have been shown to be feasible [20-22].

After the introduction of the Norwegian Health Care network, all referrals and discharge letters from hospitals in North Norway are sent electronically between primary and secondary care. Despite this improvement, poor communication often results in inefficiencies and unsatisfactory outcomes. Studies show that both referral and discharge letters were missing vital medical information, referral letters to such an extent that it might represent a health hazard to older patients [23]. Furthermore, poor communication results in unnecessary consultations, as well as delay in diagnosis and treatment [24-26].

This study is defined as a complex intervention, i.e. an intervention that includes several components (guidelines, electronic standardised referral and booking for outpatient surgery) [27]. This trial is directed at health professional behaviour with a strategy for implementing guidelines and computerised decision support. According to Campbell et al., it is useful to consider the process of development and evaluation of such interventions as having several distinct phases (theory, modelling, exploratory trial, definitive randomised controlled trial and long-term implementation). In this trial we will follow these phases, where this study protocol describes these phases and how we will conduct the definitive randomised trial.

An important decision in trials of complex interventions is whether health outcome needs to be assessed. In this "one-stop trial", all patients are receiving the same treatment (day case surgery) in both trial arms. In our opinion it is therefore sufficient to investigate the possible change of health care behaviour, decreased waiting time and increased cost benefit induced by the intervention.

In this trial we chose a RCT design involving nonpharmacologic treatment based upon the CONSORT guidelines [7]. We found that a cluster randomised trial was not feasible, to avoid bias among the clusters and difficulties in estimating within and between cluster components of variance. A cluster randomised trial would also increase the sample size [28-30]. The primary consequence of adopting a cluster randomised design is that it is not as statistically efficient and has a lower statistical power than a randomised trial of equivalent size [31].

A trial has reported a negative outcome of implementing referral guidelines among GPs for patients with lower bowel symptoms. In this study GPs were offered an electronic interactive referral pro forma. This interactive electronic referral was not integrated in the electronic health record [32]. However, in the present one-stop trial guidelines, electronic referral and booking will be fully integrated in the GP's electronic health records. This will decrease workload and probably increase enthusiasm among the GPs participating in the project.

## Competing interests

The authors declare that they have no competing interests.

## Authors' contributions

The study was initiated by KMA and R–OL. KMA is the grant holder. All authors participated in the study concept and design. RJ provided expertise in randomised controlled trials, statistics and health economics. R–OL, KMA, AR and BV developed guidelines for referral. KMA and R–OL developed the electronic referral forms. All authors reviewed and approved the final version of this manuscript.

## Pre-publication history

The pre-publication history for this paper can be accessed here:


